# 

**Published:** 1870-12

**Authors:** 


### MARRIED.

CORSON—SHURTE.—In Portsmouth, Ohio, Tuesday evening, Dec. 27th, at the residence of Capt. N. W. Evans, by the Rev. Dr. Pratt, Dr. R Corson to Repta Shurte, both of Middletown, Ohio.

### SPECIAL NOTICES.

### WATT & WILLIAMS' IMPROVED METALLIC BASE

Is giving unparalleled satisfaction, both to patient and operator, wherever it is used. It is as much cleaner than rubber work as rubber is cleaner than soldered silver work. It corrodes no more in the mouth than 18 or 19 carat gold—not as much in some mouths—and makes the strongest work ever offered to the profession. Every machine is only as strong as its weakest part; and though this metal is not as strong as iron, or even as aluminum, yet it is stronger than the porcelain of the teeth, and gives them a more evenly support than any other material used. It is as far superior to the other metallic bases in the market as they are superior to tinners' solder. When properly made it gives better satisfaction in upper than in lower plates. Patients who have worn rubber plates are perfectly delighted with the cool, pleasant sensation in the mouth, afforded by wearing a plate of this. And now, with Geo. Watt & Co.'s new flask, it is as easy to make it as to mold bullets—no office rights, no injunctions, no explosions of vulcanizers, no fumes of sulphur, and no Josiah Bacon connected with it! A complete equipment, aside from material, costs $1.50. The way to compete with low-priced work is to give your patients something better.

☞ Codman & Shurtleff's Spittoons, the neatest thing out, for sale by GEO. WATT & CO.

### GOLD FOIL.

GEO. WATT & CO. have just received a large supply of the most popular Gold Foils, of the most desired numbers, from 4 to 120; also, Pack's Eureka Gold, Watt's Crystal Gold, etc.; and they invite special attention to a new CHEMICALLY PURE GOLD FOIL of their own manufacture, pronounced the best ever tried by our most competent operators. All the above sold at the lowest cash rates; and special inducements to purchasers by the ounce.

### New Orleans Dental College

***Cor. of Carondelet & Perdido Sts.***

---

### FOURTH SESSION—1870-71.

---

***FACULTY.***

JAS. S. KNAPP, D. D. S.,
Professor of Theory and Practice.

ANDREW F. McLAIN, D. D. S., M. D.,
Professor of Institutes of Dentistry.

WM. S. CHANDLER, D. D. S.,
Professor and Clinical Instructor of Dental Surgery.

CHAS. E. KELLS, D. D. S.,
Professor of the Science of Dental Mechanism.

S. MINTER ANGELL, M. D.,
Professor of Anatomy and Physiology.

J. S. HARRISSON, A. M , M. D.,
Professor of Materia and Medical Jurisprudence.

JOHN G. ANGELL, D. D. S.,
Clinical Instructor of Dental Mechanism and
Adjunct Prof. Operative Dental Surgery.

S. P. CUTLER, M. D., D. D. S.,
Professor of Chemistry, Metallurgy, Microscopy and Histology

---

DEAN OF THE FACULTY, - - - JAMES S. KNAPP, D. D. S
*Office, No. 15 Baronne Street, New Orleans.*

---

The Fourth Regular Course of Lectures of the New Orleans Dental College for the Session of 1870-71, will commence on the 15th of November, and continue four months.

The superior advantages of this location for a Dental College should not be forgotten by students who live in a colder climate, and who by coming here will avoid the severity and rigors of a Northern winter, and enjoy four months of comparatively mild and pleasant weather. To those in delicate health this is an inducement of no inconsiderable importance.

**Professor's Tickets, for full course, - - - - - - $100 00**
**Matriculation (paid once), - - - - - - - - - - - - - 5 00**
**Diploma Fee, - - - - - - - - - - - - - - - - - - - 30 00**

Board and lodging can be procured as cheaply in New Orleans as any large city in this country.

For further information address the Dean of the Faculty,

J. S. KNAPP, D. D. S,
Office No. 15 Baronne St., New Orleans

Or the Secretary,

PROF. A. F. McLAIN, D. D. S., M. D.
Acting Dean, Box 834

New Orleans, May 21, 1870.] May,'70,8

### THOMAS DENNIS,

Manufacturer of

### SUPERIOR GOLD FOIL

*199 Main Street, Buffalo, N. Y.*

Many years' experience in the refining and beating Gold enables me to offer a superior Foil,—soft, and also adhesive, uniform in its working and giving satisfaction wherever used. A trial is solicited.

| | | |
|---|---|---|
| *Per Ounce,* | - - - - | *$36.00.* |
| *One-eighth Ounce,* | - - - | *4.75.* |

THOMAS DENNIS,
199 Main St., BUFFALO

For sale by dealers and by

N. J. CHAPIN,

37 Walnut St., CINCINNATI, O.

May,'70,1y

### PHILADELPHIA DENTAL COLLEGE.

108 North Tenth St., above Arch.

SEVENTH ANNUAL SESSION, 1869-70.

### FACULTY.

C. A. KINGSBURY, M.D., D.D.S.,
Professor of Operative Dentistry and Dental Histology.
J. H. McQUILLEN, M.D., D.D.S.. Professor of Physiology.
ALBERT R. LEEDS, A. M., Professor of Chemistry.
WM. C. HEAD, D.D.S., Demonstrator of Operative Dentistry.
D. D. SMITH, D.D.S., Professor of Mechanical Dentistry and Metallurgy.
J. FOSTER FLAGG, D.D.S., Professor of Dental Pathology & Therapeutics.
HARRISON ALLEN, M.D., Professor of Anatomy and Surgery.
WM. P. HENRY, D.D.S., Demonstrator of Mechanical Dentistry.

The Dispensary and Laboratory of the College will be open and preliminary lectures de livered by one of the Professors, every day during the latter half of October, commencing on the 19th inst. The regular course of instruction will commence on the 1st of November, and continue until the close of the ensuing February. Every opportunity will be afforded in the Clinic and Laboratory for obtaining a *practical* knowledge of Operative and Mechanical Dentistry.

### FEES.

| | |
|---|---|
| Matriculation (paid but once) - - - - - - | $5 00 |
| Tickets for the *entire* Course, including the Demonstrators' | 100 00 |
| Diploma - - - - - - - - - - - - | 30 00 |

For further particulars address J. H. McQUILLEN, DEAN,
No. 1112 Arch Street Philadelphia.

// BALTIMORE COLLEGE OF DENTAL SURGERY

THIRTY-FIRST ANNUAL SESSION, 1870-71.

### FACULTY.

THOMAS E. BOND, A.M., M.D., Prof. of Pathology and Therapeutics.
PHILIP H. AUSTEN, A.M., M.D., D.D.S., Prof. of Dental Science and Mechanism.
FERDINAND J. S. GORGAS, A.M., M.D., D.D.S., Prof, of Dental Surgery
A. SNOWDEN PIGGOT, A.M., M.D., Prof. of Chemistry and Metallurgy.
RUSSELL MURDOCH, A.M., M.D., Prof. of Anatomy.
HENRY REGINALD NOEL, A. M., M. D., Professor of Physiology.
HUGH McGINNIS GRANT, D.D.S., Demonstrator of Operative Dentistry.
THOMAS SOLLERS WATERS, D.D.S., Demonstrator of Mechanical Dentistry.
CLAUDE BAXLEY, M. D., Demonstrator of Anatomy.

The *Thirtieth* Annual Session will commence on the fifteenth of October, 1870, and continue until March, 1871.

| | |
|---|---|
| Lecture and Demonstration Fees, - - - | $115 |
| Matriculation Fee (paid only once) - - - - | 5 |
| Diploma Fee. - - - - - - - - | 30 |

Graduates of the Baltimore College of Dental Surgery can graduate at the Washington University of Medicine, Baltimore, on attending *one course* of Medical Lectures.

For information, address

F. J. S. GORGAS, M.D., Dean of the Faculty.

March. 8'6 — *No.* 43 *Hanover St., Baltimore, Md.*

### Pennsylvania College of Dental Surgery.

THE FIFTEENTH ANNUAL SESSION, 1870-71.

### FACULTY.

T. L. BUCKINGHAM, D D.S., Prof. of Chemistry.
E. WILDMAN, M.D., D.D.S., Prof. of Mechanical Dentistry and Metallurgy.
G. T. BARKER, D.D.S., Prof. of Dental Pathology and Therapeutics.
JAMES TRUMAN, D. D. S., Prof. of Dental Histology and Operative Dentistry.
JAMES TYSON, M.D., Professor of Physiology and Microscopic Anatomy.
J. EWING MEARS, M.D., Prof. of Anatomy and Surgery.
J. M. BARSTOW, D.D.S., Demonstrator of Mechanical Dentistry.
ELIHU R. PETTIT, D.D.S., Demonstrator of Operative Dentistry.

The regular Course will commence on the first Monday of November, and continue until the first of March ensuing. During September the Laboratory will be open, and Preliminary lectures will be delivered during October. A Clinical Lecture delivered every Saturday by one of the Professors. The most ample facilities are furnished for a thorough course of practical instruction.

Tickets for the Course, Demonstrators' Tickets included, $100. Matriculation Fee, $5. Diploma Fee, $30.

Publishers of the "Dental Times."

For further information, address T. L. BUCKINGHAM, DEAN,
1206 *Vine Street, Philadelphia.*

### The Dental Register

### OF THE WEST.

*Issued Monthly, at $3 a Year in Advance.*

DEVOTED TO THE INTERESTS OF THE DENTAL PROFESSION.

EDITED BY J. TAFT AND G. WATT.

The volume commences in January and closes in December.

The Register being issued monthly is always fresh, therefore indispensable to the practitioner who desires to keep posted up with the new inventions and improvements which are daily developed. Neither expense nor effort will be spared to give value and variety to its contents. Articles will be illustrated with well executed engravings whenever they will add to the interest or assist in explanation.

Its extensive circulation and frequency of issue makes it one of the BEST DENTAL ADVERTISING MEDIUMS in the United States.

RATES OF ADVERTISING.

One page, 1 year, $50. Half page, 1 year, $30. Quarter page, or card, 1 year $20.

All advertising bills payable in advance, or at furthest after the issue of the first number containing the advertisement. All transient advertisements to be paid for in advance.

☞ CONTRIBUTIONS SOLICITED.

Address, J. TAFT, or "DENTAL REGISTER," Cincinnati Ohio.

Business Communications to

**J. TAFT, Publisher,**

No. 117 West Fourth St.

